# D-cycloserine in Prelimbic Cortex Reverses Scopolamine-Induced Deficits in Olfactory Memory in Rats

**DOI:** 10.1371/journal.pone.0070584

**Published:** 2013-08-02

**Authors:** Marta Portero-Tresserra, Paula Cristóbal-Narváez, Margarita Martí-Nicolovius, Gemma Guillazo-Blanch, Anna Vale-Martínez

**Affiliations:** Departament de Psicobiologia i Metodologia de les Ciencies de la Salut, Institut de Neurociencies, Universitat Autonoma de Barcelona, Bellaterra, Barcelona, Spain; Université Lyon, France

## Abstract

A significant interaction between N-methyl-D-aspartate (NMDA) and muscarinic receptors has been suggested in the modulation of learning and memory processes. The present study further investigates this issue and explores whether d-cycloserine (DCS), a partial agonist at the glycine binding site of the NMDA receptors that has been regarded as a cognitive enhancer, would reverse scopolamine (SCOP)-induced amnesia in two olfactory learning tasks when administered into the prelimbic cortex (PLC). Thus, in experiment 1, DCS (10 µg/site) was infused prior to acquisition of odor discrimination (ODT) and social transmission of food preference (STFP), which have been previously characterized as paradigms sensitive to PLC muscarinic blockade. Immediately after learning such tasks, SCOP was injected (20 µg/site) and the effects of both drugs (alone and combined) were tested in 24-h retention tests. To assess whether DCS effects may depend on the difficulty of the task, in the STFP the rats expressed their food preference either in a standard two-choice test (experiment 1) or a more challenging three-choice test (experiment 2). The results showed that bilateral intra-PLC infusions of SCOP markedly disrupted the ODT and STFP memory tests. Additionally, infusions of DCS alone into the PLC enhanced ODT but not STFP retention. However, the DCS treatment reversed SCOP-induced memory deficits in both tasks, and this effect seemed more apparent in ODT and 3-choice STFP. Such results support the interaction between the glutamatergic and the cholinergic systems in the PLC in such a way that positive modulation of the NMDA receptor/channel, through activation of the glycine binding site, may compensate dysfunction of muscarinic neurotransmission involved in stimulus-reward and relational learning tasks.

## Introduction

It has been extensively demonstrated that the cholinergic and glutamatergic systems are involved in cognitive processes, and some lines of evidence suggest an interaction between muscarinic and *N*-methyl-d-aspartate receptors (NMDARs) in the regulation of learning and memory [1], which has mainly been assessed in the hippocampus. Thus, in vitro studies showed that the activation of muscarinic receptors increased the probability of generating NMDA-dependent long-term potentiation (LTP) [2]. Moreover, the acute application of memantine, a non-competitive NMDA receptor (NMDAR) antagonist approved for the treatment of Alzheimer’s disease, caused a significantly enhanced synaptic transmission in hippocampal slices that was blocked by the muscarinic antagonist scopolamine (SCOP) [3].

Behavioral pharmacological studies also suggest that these systems may work interactively. Firstly, systemic concomitant administration of ineffective doses of muscarinic and NMDAR antagonists produced amnesic effects in several tasks, such as spatial mazes [4], [5], contextual fear conditioning [1], inhibitory avoidance [6], [7] and a visual recognition memory task [8]. Recent studies confirmed significant deficits when sub-threshold doses were co-administered intracerebrally. Specifically, injections in the medial septum or CA1 [9] or the ventral tegmental area [10] induced amnesia in inhibitory avoidance. Secondly, systemic pre-learning infusions of d-cycloserine (DCS), a NMDAR partial agonist at the glycine modulatory site that enhances memory processes [11], [12], [13], [14], [15], attenuated SCOP-induced deficits in the acquisition of spatial tasks [16], [17], [18], [19]. Additionally, it has been shown that acute application of memantine also reversed SCOP-induced learning impairments in the water maze [3]. In non-spatial paradigms, DCS injected prior to retention reduced the negative effects of SCOP on brightness discrimination [20] and visual recognition [21]. As for intracerebral studies, early research suggested that the hippocampus may be involved in the muscarinic/NMDA interaction as reversal, with DCS, of SCOP-induced deficits in spatial working memory was found when both drugs were injected into the hippocampus [22], [23]. Furthermore, a recent report showed that injections of NMDA into the medial septum, a main hippocampal afferent, reduced SCOP-induced amnesia in inhibitory avoidance [24].

Nevertheless, a better understanding is needed of the critical brain structures in which the proposed systems interaction may occur in modulating memory. Therefore, in the present study we evaluated the effects of SCOP and DCS injected into the prelimbic cortex (PLC) as the previous literature suggests that this cortical region may be a suitable candidate. In this regard, a homogenous distribution of both glutamatergic and acetylcholinergic innervation has been described in the PLC [25]. It has also been shown that muscarinic agonists modulated the amplitude of the excitatory postsynaptic potentials, mediated by glutamate receptors, in 100% of the PLC neurons tested [26]. Previous reports also demonstrated that intra-PLC administration of SCOP disrupted memory assessed in associative paradigms based on olfaction, such as social transmission of food preference (STFP) [27], [28] and odor discrimination (ODT) [29] tasks. This is especially relevant as smell loss and pathological involvement of the olfactory pathways are present in the formative stages of neurodegenerative diseases [30]. Moreover, the ODT paradigm is sensitive to the beneficial effects of intra-PLC DCS, as an acute pre-learning treatment improved the performance of non-lesioned rats [31] and rats with thalamic lesions [32]. Both learning tasks are naturalistic appetitive forms of associative memory and independent of spatial information [33], but differ in some of the structures and underlying memory systems on which they rely. ODT mostly depends on a network of closely related brain regions, particularly in the prefrontal cortex (PLC, infralimbic, orbital) and the amygdala [34], [35] and STFP is related to the prefrontal cortex [28], [36] and amygdala [37] but also to the hippocampal formation [33], [38], [39], [40], [41].

The purpose of the present study was to examine whether NMDAR activation in the PLC may compensate dysfunction of PLC muscarinic neurotransmission assessed in two olfactory learning tasks with a differential involvement of the hippocampus. As in previous studies [28], [29], [31], [32], an acute DCS treatment was administered 20 min before ODT and STFP learning, SCOP was injected immediately afterwards, and memory was assessed in a subsequent 24-h retention test (experiment 1). As in the standard 2-choice STFP paradigm (experiment 1) DCS did not facilitate memory in SCOP-untreated rats and the reversion of SCOP-induced deficits was less conspicuous than in the ODT, a second experiment was performed in which the number of choice alternatives in the STFP test was increased to three. A test containing more response options may elude a potential ceiling effect in the percentage of food preference as it allows a wider scope for observing performance improvements due to DCS administration. Moreover, the inclusion of more distracter foods may increase the task difficulty and the engagement of the frontal cortex [42], [43]. This structure, including the PLC, is particularly related to cognitive flexibility and behavioral inhibition in the decision-making process (during the selection response) [44], [45], which may suggest that potentiating NMDA transmission would more effectively enhance memory in demanding conditions.

## Materials and Methods

### Ethics Statement

All procedures were carried out in compliance with the European Community Council Directive for care and use of laboratory animals (86/609/European Community Council) and with the Generalitat de Catalunya’s authorization (Diari Oficial de la Generalitat de Catalunya 2450 7/8/1997, Departament d'Agricultura Ramaderia i Pesca protocol number 5959).

### Experiment 1: ODT and Two-choice STFP

#### Subjects

Forty-six Wistar male rats belonging to our laboratory’s breeding stock were used (mean age = 97.7d, SD = 4.28; mean weight = 378.95g, SD = 26.72 at the beginning of the experiment). An additional set of 41 male Wistar rats (mean age = 57.45 d, SD = 4.68; mean weight = 259.54 g, SD = 42.45 at the beginning of the experiment) served as demonstrator subjects in the STFP task. All the rats were single-housed in 50×22×14 cm plastic-bottomed, sawdust-bedded cages in a room controlled for temperature (20–22°C) and humidity (40%–70%). The rats were maintained on a 12 h light-dark cycle (lights on at 8:00 a.m.), with experiments performed during the light phase of the cycle. Rat-chow pellets (Scientific Animal Food & Engineering, Augy, France) and water were provided *ad libitum* except during habituation, acquisition and test sessions, in which the rats were submitted to a food restriction schedule (12 g/d to maintain body weight at 85% of their free-feeding weight). The animals were handled on a daily basis for 5 min and restrained for 2 min to accustom them to the injection procedure.

#### Surgery

**Figure 1:**
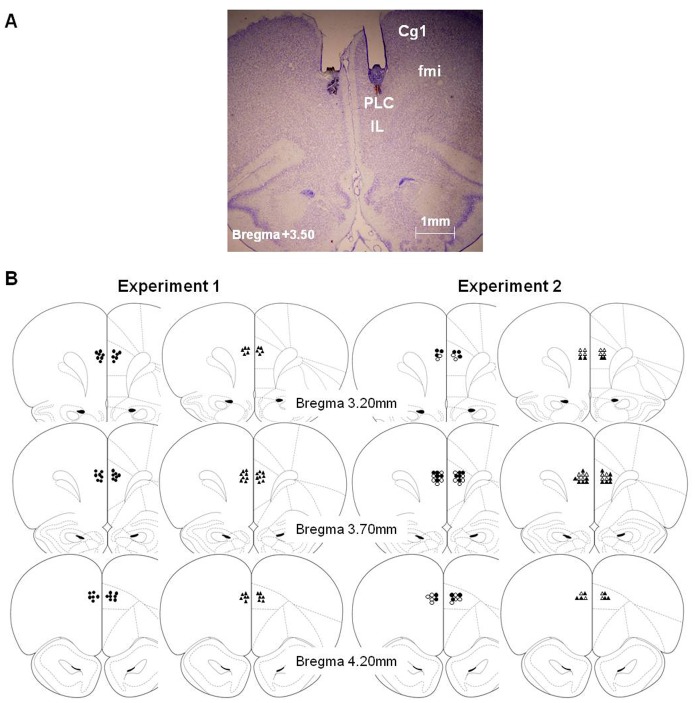
Histology. A) Photomicrographs of Cresyl violet staining at the level of the PLC area (AP, 3.50 mm anterior to bregma) showing the cannula track and the micro-injector tip of a representative subject [Cg1, cingulate cortex area 1; Fmi, forceps minor of the corpus callosum; IL, infralimbic cortex; PLC, prelimbic cortex] (B) Micro-injector tip placements throughout the rostral-caudal extent of the PLC (Paxinos and Watson, 1997) in experiment 1 (DCS and DCS+SCOP are represented by filled circles; VEH and SCOP by filled triangles) and experiment 2 (VEH is represented by empty circles; DCS by filled circles; SCOP by empty triangles; DCS+SCOP by filled triangles).

Animals were anesthetized and underwent stereotaxic implantation of bilateral chronic double-guide cannulae into the PLC following procedures explained in detail elsewhere [28], and all efforts were made to minimize suffering. Each guide cannula comprised two 26-gauge metal tubes projecting 2.9 mm from the pedestal (Plastics One, Bilaney Consultants GMBH, Düsseldorf, Germany.). The stereotaxic coordinates for implantation in the PLC were (Fig. 1A): AP, +3.5 mm from bregma; ML, ±0.6 mm from midline; and DV, −2.9 mm from cranium surface [46]. Sterile dummy stylets (Plastics One) were placed into the cannulae to prevent occlusion. After surgery, rats were administered an antibiotic (Panolog, Novartis) and were returned to their home cages for 10 days (4 for recovery, 4 for food restriction and 2 for rehabituation) before behavioral training. During the 10-day recovery period, the rats were handled and weighed on a daily basis and the dummy stylets were changed every other day.

#### Microinfusion procedure

**Figure 2:**
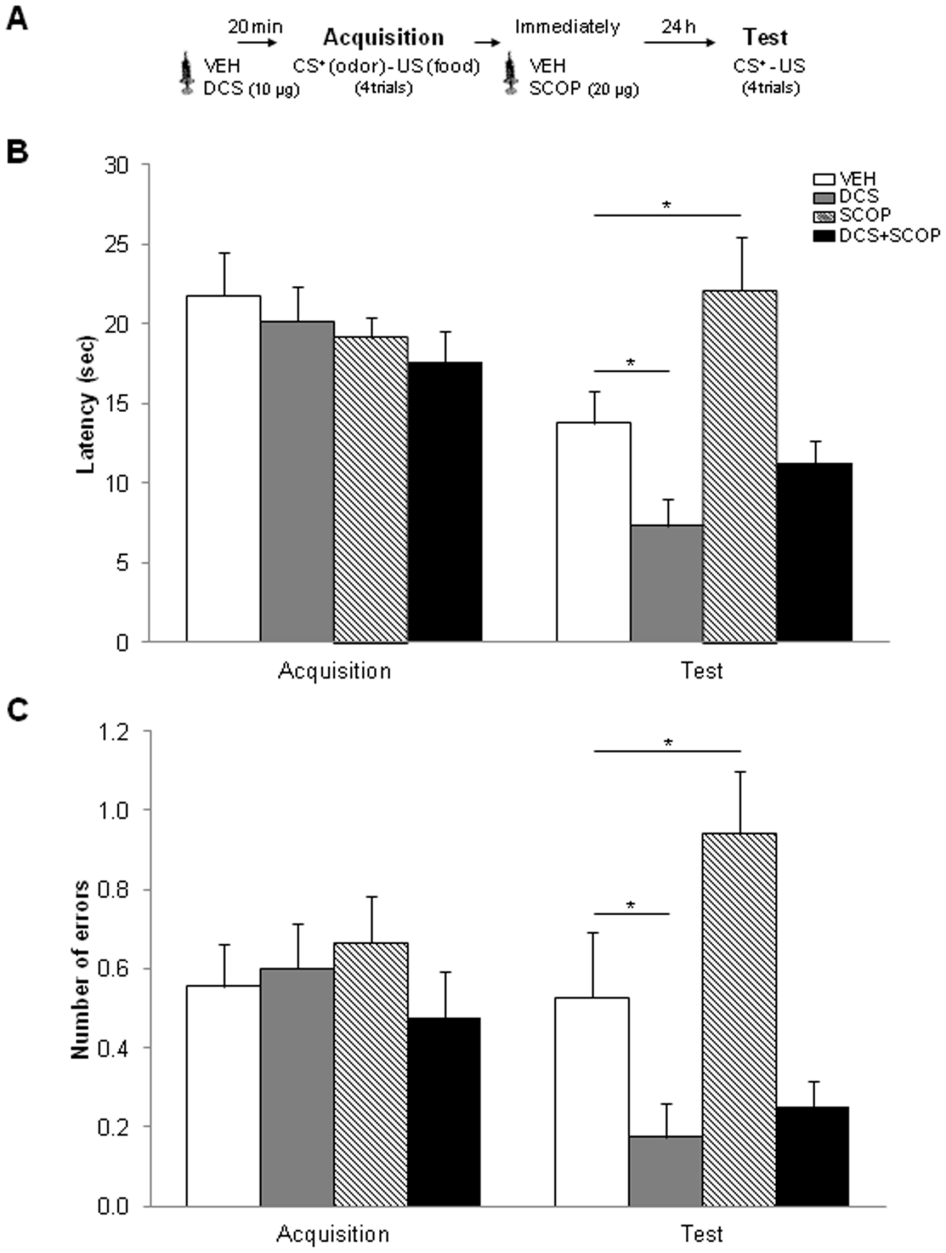
Experiment 1 (ODT). (A) The behavioral procedure used for experiment 1. (B) Latency (average of all trials) to make the correct response (±SEM) in each session. (C) Number of total errors (average of all trials) prior to making the correct response (±SEM) in each session (*p<0.05).

**Figure 3:**
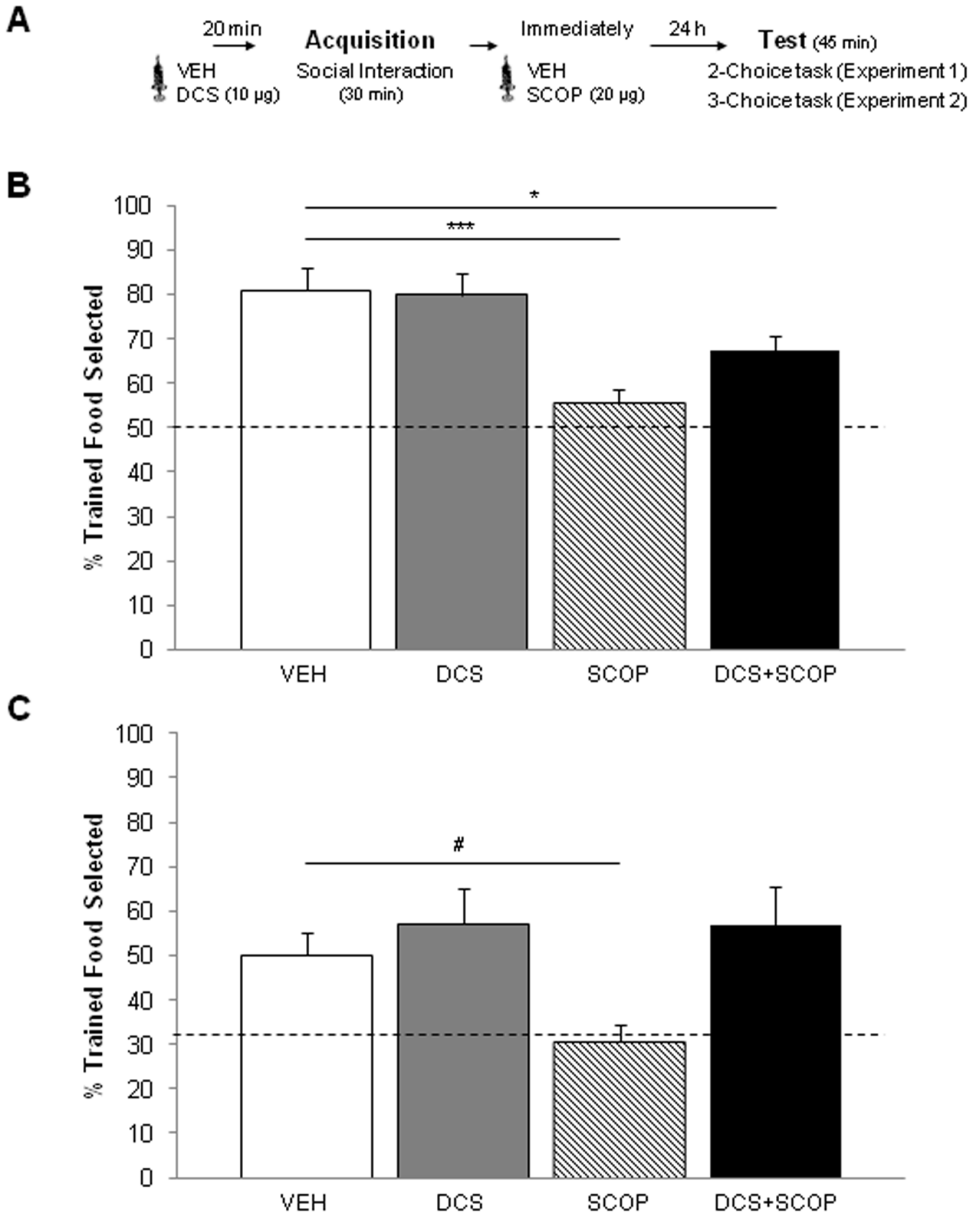
Experiments 1 and 2 (STFP). (A) The behavioral procedure used for experiment 1 and experiment 2. (B) Percentage of trained food selected, expressed as the mean percentage (± SEM) of the total amount of food consumed in the STFP two-choice test (experiment 1) and (C) the three-choice test (experiment 2) (*p<0.05,***p<0.0001, ^#^p = 0.06).

The rats received the drug infusions twenty min before (DCS/vehicle) and immediately after ODT and STFP acquisition (SCOP/vehicle) (Figs. 2A and 3A). For this purpose, they were gently restrained while the dummy stylets were removed and replaced with 33-gauge stainless-steel double injectors (Plastics One) extending 1 mm below the cannula tips. The injectors were connected by polyethylene tubing (Plastics One) to two 10-µl syringes (SGE Analytical Science, Cromlab S.L. Barcelona, Spain) mounted in an infusion pump (11 Plus Syringe Pump, Harvard Apparatus Inc., Holliston, Massachusetts, USA). DCS (Sigma-Aldrich, Madrid, Spain) and SCOP (Scopolamine Hydrobromide USP, Sigma–Aldrich Química S.A., Madrid, Spain) were dissolved in PBS (phosphate-buffered saline 0.1 M, pH 7.4) and doses of 10 µg/hemisphere (DCS) and 20 µg/hemisphere (SCOP) were administered into the PLC. The rats in the control VEH groups received vehicle (PBS) injections. The solutions were infused bilaterally in a volume of 0.5 µl/hemisphere for 2 min. The injectors were left in place for 1 min after the infusion was complete to allow for diffusion. The dose, volume and injection time of the drugs were based on previous studies in which intra-PLC DCS enhanced ODT [31], [32] and SCOP disrupted ODT and STFP memory [28], [29].

#### Apparatus

In the ODT, the habituation to reinforcement was performed in a plastic bottomed cage (50×22×14-cm). The training apparatus was a 60×60×40 cm square box containing three sponges with a 3-cm diameter hole cut into the centre, placed in glass slide-holders of the same size [47]. The food reinforcement used was a crispy chocolate rice breakfast cereal (Kellogg’s, Spain) that was placed at the bottom of the opening in the sponge. Each sponge was infused with an odor that was injected into all its corners. The odors, vanilla (0.3 ml), orange (0.6 ml) and anise (0.2 ml) (Vahiné, Ducros S.A., Sabadell, Spain), were previously tested in a pilot study in which the rats showed no particular preference. All behavioral sessions were recorded by a video camera (JVC, Everio Model GZ-X900) connected to a monitor.

In the STFP task, all observers were habituated, trained and tested in their own 50×22×14-cm plastic-bottomed, sawdust-bedded cages. Habituation and testing were carried out using a feeding-tray placed in the animals’ cages. The tray consisted of a black Plexiglas base (21×21-cm) with two adjacent plastic pots fixed onto the center of the base. The food (powdered rat chow) was placed in glass jars (130 ml) secured within each plastic pot. For the demonstrators, habituation and acquisition were carried out in 50×22×14-cm plastic cages in which they were allowed to eat from a glass jar mounted upon the center of a black Plexiglas base (21×10-cm). For the acquisition and test, powdered rat chow was 2.2% ground cocoa (Oxfam Fairtrade, Gent, Belgium) and/or 1% ground cinnamon (Carmencita, Alicante, Spain). All sessions were recorded by a video camera (JVC, Everio Model GZ-X900) connected to a monitor.

#### Behavioral procedure: ODT

All the animals underwent ODT and STFP in a counterbalanced way (half sample: ODT-STFP, half sample: STFP-ODT). The injections were also counterbalanced, with the subjects administered with DCS before the first task acquisition receiving VEH in the second one, and those administered with SCOP after the first task acquisition receiving VEH in the second one.

The rats were food-restricted for five days prior to three pre-surgery habituation sessions in which they were familiarized with the reinforcement and the training box. After consuming ten pieces of cereal/session, they were placed in the training box, without the reinforcement, and allowed to explore it for 15 min. Six days after surgery, rats were again food-restricted and submitted to an identical 15-min rehabituation session and a mock infusion protocol (no solutions injected) in order to minimize any stress associated with the procedure.

One day after rehabituation, ODT acquisition was carried out in a single four-trial session (Fig. 2A), according to procedures previously described [31]. The reinforcement (chocolate rice cereal placed at the bottom of the opening in the target sponge) was associated with the same odor across trials, and the target odor was randomly assigned to each rat in a counterbalanced way. The sponges with the non-reinforced odors did not contain any food. Sponges were placed in any three of the four corners of the box, and the position of each odor within the box was changed for each trial according to a previously determined protocol.

The rats were placed in the training box, facing the corner with no sponge. There was a 3-min limit for the rats to find and consume the reinforcement and the inter-trial interval was 1 min. Latency before a correct response (nose-poking into the target sponge) and errors were scored. Two different errors were combined: errors of commission (nose-poking into a non-target sponge) and omissions (sniffing the target sponge not followed by nose-poking) [35]. Latencies and errors were scored by two independent judges that were blind to drugs administration.

Twenty-four hours after acquisition, the rats were tested (24h-test session) using the same procedure as in the previous acquisition session. The first test trial was not reinforced to measure memory of the previous training [35].

#### Behavioral procedure: Two-choice STFP

After 5 days of food restriction, prior to surgery, observers and demonstrators were habituated to powdered chow (Scientific Animal Food & Engineering, Augy, France) from glass jars to minimize neophobia, for 2 h on the first day, 1 h the second day and 45 min the third day. The rats were presented with food cups in feeding trays containing ground, unflavored rat-chow, in their own cages. A similar procedure was repeated 6 days after surgery for the observers (two 45-min rehabituation sessions). Subsequently, animals were food-restricted once again for 2 days before the training–testing sessions began.

The STFP acquisition and test were conducted following procedures explained elsewhere [27], [28]. Essentially, the task began when a demonstrator was allowed to eat food flavored with cocoa or cinnamon for 30 min in its own cage. Then, a demonstrator that had just eaten flavored chow was placed into the observer’s cage and the two rats were allowed to interact for 30 min. All observers were tested 24 h after STFP acquisition by placing two jars filled with odorized food, and with water available. In the STFP test, one of the jars contained the chow with the flavor that was given to demonstrators (trained food) and the other jar contained different scented chow (untrained food). The observers were allowed to eat for 45 min, after which the food jars were removed and weighed to determine the amount of food eaten from each. A preference score (Percentage of trained food) for the trained odor was calculated as follows: 100×(weight of trained food eaten/weight of all food eaten). Subjects’ behavior during the social interaction (acquisition) and testing was recorded and the number of times each observer sniffed the muzzle, body or anogenital region of the demonstrator was scored. A sniff was defined as close orientation (<2 cm) of the observer’s muzzle toward the demonstrator [47]. During the first 20 min of testing, the number of times the observer was on top of the jar with both forepaws was also scored (Jar Climbs).

#### Olfactory perception test

To rule out olfactory alterations due to the DCS and SCOP infusions, an additional olfactory perception test was conducted at the end of the experiment [48], [49] on a sample of each group (VEH: n = 8, DCS: n = 8, SCOP: n = 8 and DCS+SCOP: n = 9). Twenty-four hours before the olfactory test, the rats were habituated to butter-flavored cookies (Brambly Hedge, Denmark). Twenty min before such habituation, they were infused with DCS or PBS and with SCOP or PBS immediately after. The rats were food-restricted for 24 hours before the test, which was conducted in clean rat cages (50×22×14-cm) and a piece of cookie was buried in one of its corners. The rats were then placed in the cage, and the latency to find the buried cookie and commence eating was timed.

#### Histology

Upon completion of the behavioral testing, the rats were deeply anesthetized with an overdose of sodium pentobarbital (Dolethal, Vetoquinol SA Madrid, Spain; 200 mg/kg i.p.) and perfused transcardially with 0.9% saline followed by 10% formalin. The cannulae were carefully removed and brains were postfixed in 10% formalin for at least 24 h and then submerged in a 30% sucrose solution prior to sectioning. Coronal 40-µm sections were cut on a cryostat (Shandom Cryotome FSE, Thermo Electron Corporation, Waltham, Massachusetts, USA), mounted and stained with Cresyl violet. The sections were examined under a light microscope (Olympus BX 41; Olympus Optical CO, LTD, Tokyo, Japan) and microphotographs of the cannula placements were taken using a digital camera (Olympus DP70).

#### Data analysis

Data from ODT were submitted to a mixed analysis of variance (ANOVA; PASW v20) in which the between-factor was Group (VEH, DCS, SCOP, DCS+SCOP) and the within-factor Session (two levels: Acquisition -the average scores for the 4 trials- and Test -the average scores for the 4 trials-). The dependent variables were Latencies and total Number of errors. Post-hoc comparisons were performed between each treatment condition and the VEH group by means of Dunnett's t-tests.

The analysis of the main dependent variable in the STFP task, Percentage of trained food, was performed by means of ANOVA with the Group factor as the independent variable (VEH, DCS, SCOP, DCS+SCOP). Post-hoc comparisons were also performed between the VEH group and the remaining groups by means of Dunnett's t-tests. In addition, a one-sample t test against a constant (50) was used for each group to determine whether the percentage of trained food eaten was different from the chance level (50%). To evaluate whether all the animals had similar opportunities of learning (similar social interaction levels), we carried out an ANOVA analysis, considering Group as the independent variable and the dependent variables were sniffs of the demonstrator’s Muzzle, sniffs of the demonstrator’s Body and sniffs of the demonstrator’s Anogenital region. Pearson correlation tests were used to examine the relationship between such variables and the Percentage of trained food selected. ANOVA analyses were used to analyze Total food eaten and Jar climbs that evaluated motivation to eat and explore during the 2-choice preference test. Additional mixed analyses of variance were carried out to analyze neophobia, with the dependent variables Regular food (mean g of food eaten during the last habituation session prior to training) and New food (mean g of total food eaten, trained+untrained, during the test).

Regarding the olfactory test, an additional ANOVA analysis was applied considering Group (VEH, DCS, SCOP and DCS+SCOP) as the independent variable, and Latency in finding the buried cookie as the dependent variable.

### Experiment 2: Three-choice STFP

#### Subjects

Fifty-three male Wistar rats (mean age = 94.1 d, SD = 7.44; mean weight = 392.34 g, SD = 39.25) were used as observers and 44 rats as demonstrators (mean age = 59.02 d, SD = 6.16; mean weight 286.32 g, SD = 31.02). In experiment 2, the rats underwent surgery, microinfusion and histology using the same procedures as described for Experiment 1.

#### Apparatus

All observers were habituated and trained under the same conditions as in the STFP task in experiment 1 with the exception that in the habituation and test session the food tray contained three jars.

#### Behavioral procedure

The habituation, acquisition and testing procedures were the same as those in the STFP task from experiment 1, with the exception that, in addition to cocoa and cinnamon, 0.5% vanilla (Hacendado, Spain) was also used as a third option in the preference test.

#### Olfactory perception test

To rule out olfactory alterations, the same protocol as in experiment 1 was carried out on a sample of each group (VEH: n = 9, DCS: n = 9, SCOP: n = 9 and DCS+SCOP: n = 8).

#### Data analysis

The statistical analyses were similar to those in STFP from experiment 1, but in experiment 3 the one-sample t test was against the constant 33.3 (chance level 33.3%).

## Results

### Histology (Experiments 1 and 2)

When the experiments were completed, all the rats (except the demonstrators in experiments 1 and 2) were subjected to histological verification of correct bilateral cannula placements. Subjects were only included if their injector tips were located bilaterally within the PLC within the area delimited by the anterior cingulate and infralimbic cortices and in which no tissue damage resulting from the rate or volume of the infusions was detected (Fig. 1A). Specifically the cannulae were located along different brain coordinates from 3.20 to 4.20 mm anterior to bregma (Fig. 1B) according to the stereotaxic atlas [46]. Subjects with incorrectly implanted cannulae were excluded from behavioral data analyses (Experiment 1: n = 7, Experiment 2: n = 7). Thus, the final sample in experiment 1was made up of 39 subjects (ODT: VEH = 9, DCS = 10, SCOP = 9, DCS+SCOP = 11; STFP: VEH = 10, DCS = 10, SCOP = 8, DCS+SCOP = 11), and, in experiment 2, 44 subjects (VEH = 11, DCS = 11, SCOP = 11, DCS+SCOP = 11).

### Behavior

#### Experiment 1: ODT

The analysis of Latencies (Fig. 2B) to make the correct response showed that the Group (*F*[_3]_, [35] = 3.81; *P* = 0.018), the Session (*F*[_1]_, [35] = 19.794; *P*<0.0001) and the interaction Group×Session (*F*[_3]_, [35] = 5.698; *P* = 0.003) factors were statistically significant. Also, the analysis of the total Number of errors (Fig. 2C) demonstrated that the Group and Group×Session factors were statistically significant (*F*[_3]_, [35] = 4.896; *P* = 0.006 and *F*[_3]_, [35] = 4.158; *P* = 0.013, respectively), but not the Session factor (*F*[_1]_, [35] = 1.911; *P* = 0.176). Specifically, in the acquisition session, all the groups displayed a similar performance and between-group differences were only found in the Test session. The Dunnett t-tests demonstrated statistically significant differences in Latencies and Number of errors between the VEH group and the following groups: SCOP (*P = *0.016, *P = *0.029, respectively) and DCS (*P = *0.05, *P = *0.05, respectively), but not DCS+SCOP (*P* = 0.391, *P* = 0.119, respectively).

#### Experiment 1: Two-choice STFP

The ANOVA analysis revealed a statistically significant effect of the Group in Percentage of trained food eaten in the test, *F*[_3]_, [38] = 7.588, P<0.0001 (Fig. 3B). According to the Dunnett t-tests statistically significant differences were found between the VEH group and the SCOP (*P*<0.0001) and the DCS+SCOP (*P*<0.028) groups, but not the DCS group (*P* = 0.677). Moreover, VEH, DCS and DCS+SCOP groups significantly performed above chance level (all *t*’s>5.4, all *P*’s<0.0001), but the SCOP group showed a performance that was not statistically different from chance level (*P* = 0.098).

The analysis of the social interaction measures (Table 1) showed no statistically significant Group effects in any of the variables (Muzzle: *F*[_3,33_] = 1.547, *P = *0.223; Body: *F*[_3,33_] = 1.878, *P = *0.155; Anogenital: F[_3,33_] = 0.964, *P = *0.422). There were no statistically significant correlations between such variables and the Percentage of trained food (Muzzle: r = −0.202, *P = *0.252; Body: r = 0.194, *P = *0.272; Anogenital: r = 0.251, *P = *0.152). The analysis of the Jar climbs performed in the test (Table 1) showed that all the groups investigated both food jars to a similar degree (F[_3,33_] = 0.145, *P = *0.932) and consumed a similar amount of food (F[_3,38_] = 1.098, *P = *0.363). In the analysis of possible neophobic effects (Table 1), a mixed ANOVA analysis showed a significant effect of Food (F[__1,35__] = 6.085, *P = *0.019) but no significant effects of Group (*F*[_3,35_] = 0.268, *P = *0.848) or Group×Food interaction (*F*[_3]_, [35] = 1.784, *P = *0.168), thus demonstrating that, although the New food produced a certain neophobic response, the pattern of consumption was similar for all groups.

**Table 1:** Ancillary variables measured in STFP task in experiment 1.

	Habituation	Social Interaction			2-choice test	
	Regular food	Muzzle	Body	Anogenital	Jar climbs	Total (new) food
VEH	8.93±4.96	44.60±8.95	67.70±10.58	27.70±6.68	64.80±17.73	7.67±2.12
DCS	10.73±3.9	39.22±11.3	54.33±11.31	30.22±7.36	72.22±30.77	7.53±2.71
SCOP	8.59±2.23	34.75±8.26	68.00±17.78	26.75±6.75	68.00±14.17	8.19±2.36
DCS+SCOP	9.18±2.86	44.82±9.30	56.82±18.65	24.91±7.11	70.82±32.17	9.5±3.58

Means ± SD of the amount of regular food consumed during the last rehabituation (unodorized ground food); Means and ± SD of the number of sniffs scored during the social interaction; Means and ± SD of the number of jar climbs during the first 20 min of the 2-choice STFP test; Means and ± SD of the total amount of total odorized food eaten during the test (new food, -trained+untrained-).

#### Experiment 1: Olfactory perception test

The performance in both tasks did not seem to be related to changes in olfactory sensitivity (Table 2) since no statistically significant between-group differences were observed when the Latency to find a buried sweet-smelling cookie was analyzed 24 h after injections (F[_3,32_] = 0.756, *P = *0.528).

**Table 2:** Olfactory perception test.

	Experiment 1	Experiment 2
VEH	30.13±11.96	24.67±13.5
DCS	24.56±10.11	25.56±10.45
SCOP	32.25±12.88	25.33±11.18
DCS+ SCOP	28.15±11.64	31.75±15.63

Means ± SD of the latency (sec) to find a buried cookie in the olfactory perception test carried out in experiments 1 and 2.

#### Experiment 2: Three-choice STFP

The main analysis revealed a significant effect of the Group in Percentage of trained food eaten in the test (F[_3,43_] = 3.395 *P = *0.027) (Fig. 3C). The contrast analyses showed that the preference score of the VEH group was not statistically different from that of DCS and DCS+SCOP groups (*P* = 0.935 and *P = *0.929, respectively), and tended to be statistically higher than the SCOP group (*P* = 0.06) score. Similarly to experiment 1, and confirming the latter analysis, the VEH, DCS and DCS+SCOP rats significantly performed above chance level (all *t’s*>2.6, all *P*’s<0.025), whereas the SCOP rats showed a performance not significantly different to chance (*P = *0.505). There were no statistically significant Group effects in any of the variables measured during the social interaction (Table 3) (Muzzle: F[_3,43_] = 1.947, *P = *0.138); Body: F[_3,43_] = 1.744, *P* = 0.173; Anogenital: F[_3,43_] = 2.186, *P = *0.105). No statistically significant correlations were found between these variables and the Percentage of trained food (Muzzle: r = −0.069, *P = *0.664; Body: r = 0.260, *P = *0.096; Anogenital: r = 0.055, *P = *0.728). No statistically significant between-group differences were observed either in the total amount of food consumed during the test (*F*[_3,43_] = 1.664, *P* = 0.190) or in the Jar climbs (Table 3) (F[_3,43_] = 1.356, *P = *0.274). Mixed ANOVA analysis did not show any significant effect of Food (*F*[_1,40_] = 1.214, *P = *0.277), Group (F[_3,40_] = 1.717, *P = *0.179) or Group×Food interaction (F[_3,40_] = 0.813, *P = *0.317) (Table 3), demonstrating that SCOP or DCS did not produce neophobic reactions.

**Table 3:** Ancillary variables measured in STFP task in experiment 2.

	Habituation	Social Interaction			3-choice test	
	Regular food	Muzzle	Body	Anogenital	Jar climbs	Total (new) food
VEH	11.04±4.67	35.20±10.1	41.00±10.31	25.90±11.0	82.12±30.87	9.69±1.76
DCS	8.78±3.75	42.36±22.8	54.55±26.20	28.27±12.4	69.38±25.38	8.8±2.62
SCOP	7.99±4.39	54.4±26.24	51.10±15.27	39.3±22.13	71.91±32.34	7.22±2.71
DCS+SCOP	8.13±3.92	52.27±22.4	58.55±22.47	42.55±22.6	55.33±19.72	7.87±3.68

Means ± SD of the amount of regular food consumed during the last rehabituation (unodorized ground food); Means and ± SD of the number of sniffs scored during the social interaction; Means and ± SD of the number of jar climbs during the first 20 min of the 3-choice STFP test; Means and ± SD of the total amount of total odorized food eaten during the test (new food, -trained+untrained-).

#### Experiment 2: Olfactory perception test

Performance in the 3-choice STFP test did not seem to be related to deficits in olfactory sensitivity (Table 2) since no statistically significant between-group differences were observed in the test (*F*[_3]_, [34] = 0.553, *P = *0.650).

## Discussion

The current research shows that potentiating NMDAR function in the PLC by DCS may attenuate mnemonic deficits induced by muscarinic receptor antagonism in two olfactory learning paradigms, a stimulus-reward task and a relational memory task [38], [50], [51], which share some underlying structures, such as the PLC, but not others, such as the hippocampus [28], [33], [34]. Such findings cannot be attributed to alterations in olfactory perception, social investigation, neophobic responses or motor activity since DCS and SCOP infusions, alone or in combination, did not show any effect in the olfactory sensitivity test or the ancillary variables scored during social interaction and food preference testing. Likewise, the counteraction of SCOP-induced deficits was observed in other learning paradigms (see Introduction section) using DCS administration, which also attenuated mnemonic deficits induced by the blockade of other neurotransmission systems, such as NMDA [52]. Moreover, DCS has been able to revert memory deficits associated to aging [53], [54], stress [55], traumatic brain injury and hipocampal or medial septal lesions [56], [57], [58], [59].

In the present experiments, the reversion effect of the pre-training DCS treatment was highly noticeable on the ODT in which the DCS+SCOP group performance in the 24 h drug-free test did not significantly differ from that of the VEH, in contrast to the poorer performance by the SCOP rats in terms of both latencies and errors. This agrees with previous findings demonstrating that pre-acquisition intra-PLC DCS rescued ODT memory impairment induced by parafascicular lesions [32]. As for the STFP, DCS microinfusion also ameliorated the SCOP-induced deficits since DCS+SCOP rats showed a preference score superior to the chance level, like the VEH and DCS rats and unlike the SCOP rats which performed around 50% (2-choice test) or 33.3% (3-choice test). Nonetheless, DCS appeared to be more effective in the 3-choice version of the task because the DCS+SCOP group did not significantly differ from the VEH group, in contrast to the 2-choice paradigm. A possible explanation for such an outcome is that the prefrontal cortex may be more actively engaged in the STFP task when its difficulty is increased and decision-making is arduous [42], [43], which would agree with the proposed role of the prefrontal cortex in a variety of processes associated with executive function, including decision-making [60]. This would suggest that challenging tests (e.g. involving several choice alternatives) may be a more appropriate way to evaluate promnesic effects [61].

The data presented here also show that a single injection of DCS in the PLC prior to learning improved the odor-reward task in SCOP-untreated rats, as the group treated with DCS alone performed significantly better than the VEH group. This effect replicates previous findings indicating that DCS-treated rats committed significantly fewer errors in a 24 h ODT test [31] and corroborates that NMDARs in the PLC modulate ODT memory formation since microinfusion of the NMDAR antagonist APV into the PLC (but not the hippocampus) impaired an ODT retention test [35]. Although the outcome of DCS only affecting the 24 h test, as opposed to acquisition, may be unexpected, it rules out the possibility of a state-dependent learning situation. Moreover, it has previously been shown that PLC SCOP infusions or thalamic lesions carried out prior to ODT acquisition may result in delayed effects [29], [49]. As for the STFP task, involvement of the NMDA receptors has previously been demonstrated in experiments administering NMDAR antagonists systemically or in the hippocampus, inducing amnesic effects [62], [63]. In contrast, and also in opposition to the ODT results, our research shows that the positive modulation of PLC NMDAR did not produce any significant effect in social memories transmitted by odorous stimuli in SCOP-free rats.

Such findings suggest that DCS may have differential effects depending on the nature of the learning paradigm and may be interpreted as DCS enhancing implicit or procedural tasks, such as ODT, but its facilitative influence on relational paradigms, such as STFP, was limited. In this regard, there is evidence showing, on the one hand, that DCS facilitated ODT [15], conditioned fear responses [64], [65], [66], conditioned flavor-taste preference and conditioned-taste aversion [13], [67], or procedural learning in humans [68]. On the other hand, no facilitative effects of DCS administration were found in the retention of Morris water maze (MWM) learning in rodents [69], [70], or declarative word-pair learning in humans [68]. Nevertheless, other reports point to the facilitation of hippocampal-dependent paradigms, such as MWM [11], [53], [71], radial arm maze [72], linear maze [73], object-location [74], trace eye blink conditioning [75], an episodic-like memory task [61] and item-category associations [76]. Indeed, the view that distinguishes declarative/hippocampal tasks from procedural/non hippocampal tasks has been challenged and it has been suggested that multiple brain regions involved in learning are linked to each other in a coordinated way, rather than working in isolation and competing for control over behavioral output [77].

The inconsistent effects of DCS on learning and memory may be attributable to additional factors observed in the different experiments, such as dissimilar drug doses and injection timings, test protocols, rat strains or species, and/or ages. In view of such evidence, our results may potentially contain some limitations in the STFP task. The DCS dosage, for example, may not have been optimal, which is an important factor in that the therapeutic window for DCS to enhance human fear memory extinction has been reported as narrow [78]. In this respect, although the previous studies testing intracerebral DCS administration used the same dosage (10 µg/site) [15], [31], [79], [80], [81], higher doses might have been more appropriate to find enhancing effects in VEH rats. Indeed, it has been reported that only a higher dose of systemic DCS was able to promote episodic-like memory [61], although lower doses potentiated memory in non-relational aversive paradigms [82]. However, the use of higher doses of DCS could not induce outstanding facilitative effects since a reversed U-shaped dose-response curve has been described in behavioral and electrophysiology studies [83], [84], [85]. Additionally, other brain areas besides the PLC may be more sensitive to intracerebral DCS administration, such as the hippocampal formation, which has been clearly involved in the consolidation of STFP [33], [38] and other relational tasks.

The present study also confirms that the blockade of cholinergic muscarinic receptors in the PLC notably damaged memory in ODT and STFP. Such findings corroborate previous data showing that intra-PLC post-training SCOP infusions disrupted memory tests performed one day after ODT or STFP acquisition [27], [29], [28]. Additional examples of SCOP-induced deficits can be found when the drug is injected in other brain regions, e.g. the basolateral amygdala, which also interrupted STFP [37], the hippocampus impairing contextual fear conditioning [86], the cingulated and insular cortices disrupting inhibitory avoidance [87], [88] or the perirhinal cortex decreasing recognition memory [89]. Although the administration of muscarinic receptor antagonists has frequently been considered a pharmacological model for cholinergic cognitive impairment mimicking some of the features of neurodegenerative disorders [90], the use of SCOP remains controversial due to its wide mode of action and spectrum of behavioral effects [91]. In this respect, it has been suggested that selective M1 antagonists may constitute a relatively more valid pharmacological model of cognitive impairment as they are likely to affect cognitive function in a relatively more specific manner [90]. Nevertheless, importantly to the present research, the fact that SCOP impairs social memory [27], [28], [92], combined with the clinical observation of reduced social contacts in dementia patients, may suggest that social behavior based-tasks that are sensitive to muscarinic blockade, such as the STFP, may offer a relevant approach with translational value for experimental models of cognitive dysfunction.

As for the mechanisms of action, DCS effects have been interpreted in terms of synaptic plasticity modulation [93], [94], considering that it is capable of enhancing NMDAR-dependent synaptic potentials and LTP in the CA1 hippocampal field of control adult and old rats [95], [96], [97]. Similarly, DCS reinstated hippocampal LTP and improved neurological and learning recovery in brain-damaged mice [59] and neural cell adhesion molecule-deficient mice [98]. It may be complex, however, to understand why the combination of DCS and SCOP, with different pharmacological mechanisms, demonstrated a balancing or compensatory effect. Some data indicate that cholinergic actions may be mediated via the regulation of NMDARs, whose properties enable many forms of indirect modulation [99]. In particular, the stimulation of muscarinic receptors is known to facilitate the activation of NMDARs causing a long-lasting facilitation of excitatory postsynaptic potentials [2]. Also, a recent study has shown that the synergistic coactivation of muscarinic and glutamatergic receptors is essential for long-lasting LTP and that cooperation between such receptors is needed to induce BDNF-dependent long-lasting memory storage [100]. Most of these actions have been described in the hippocampal region, although they may also take place in neocortical regions such as the medial prefrontal cortex [101], [102]. In this context, it has been suggested that cholinergic and NMDA receptors jointly modulate the electrophysiological functioning of cortical cells [54], [103]. Thus, the activation of muscarinic receptors has been reported to increase glutamate release, which positively modulates neuronal activity in cortical pyramidal cells [104], [105].

Therefore, although our results do not fully demonstrate an interactive relationship between the glutamate and acetylcholine systems in learning and memory modulation, they are in line with other studies suggesting such a relationship (see Introduction section). Consequently, in the present experiments, SCOP may have disrupted potential plasticity mechanisms [106] in the PLC, which were possibly restored by DCS administration, and thus improved ODT and STFP memory. Although such tasks are based on olfactory cues, similar effects may well be found in mnemonic tasks depending on different sensory modalities. This is suggested by the fact that the PLC has been related, for instance, to the reversal learning of associative visual discrimination tasks [107]. This would also indicate that the PLC not only participates in specific associative memory but also in more general aspects of cognitive demand, such as behavioral flexibility, which may be important in processing information for different kinds of memory [108]. Further research would also need to be performed in order to determine the precise mechanisms underlying the interactive process between neurotransmitter systems and the most effective doses and sites of action of DCS to facilitate different memory paradigms and thus contribute to accelerating the effectiveness of cognition-enhancing therapies.
